# The Heat Shock Protein 40-Type Chaperone MASH Supports the Endoplasmic Reticulum-Associated Degradation E3 Ubiquitin Ligase MAKIBISHI1 in *Medicago truncatula*

**DOI:** 10.3389/fpls.2021.639625

**Published:** 2021-02-23

**Authors:** Marie-Laure Erffelinck, Bianca Ribeiro, Lore Gryffroy, Avanish Rai, Jacob Pollier, Alain Goossens

**Affiliations:** ^1^Department of Plant Biotechnology and Bioinformatics, Ghent University, Ghent, Belgium; ^2^VIB Center for Plant Systems Biology, Ghent, Belgium

**Keywords:** chaperone, E3-ubiquitin ligase, endoplasmic reticulum, 3-hydroxy-3-methylglutaryl-CoA reductase, jasmonate, protein quality control, RING membrane-anchor protein, triterpene saponin

## Abstract

Jasmonates (JA) are oxylipin-derived phytohormones that trigger the production of specialized metabolites that often serve in defense against biotic stresses. In *Medicago truncatula*, a JA-induced endoplasmic reticulum-associated degradation (ERAD)-type machinery manages the production of bioactive triterpenes and thereby secures correct plant metabolism, growth, and development. This machinery involves the conserved RING membrane-anchor (RMA)-type E3 ubiquitin ligase MAKIBISHI1 (MKB1). Here, we discovered two additional members of this protein control apparatus via a yeast-based protein–protein interaction screen and characterized their function. First, a cognate E2 ubiquitin-conjugating enzyme was identified that interacts with MKB1 to deliver activated ubiquitin and to mediate its ubiquitination activity. Second, we identified a heat shock protein 40 (HSP40) that interacts with MKB1 to support its activity and was therefore designated MKB1-supporting HSP40 (MASH). *MASH* expression was found to be co-regulated with that of *MKB1*. The presence of MASH is critical for MKB1 and ERAD functioning because the dramatic morphological, transcriptional, and metabolic phenotype of *MKB1* knock-down *M. truncatula* hairy roots was phenocopied by silencing of *MASH*. Interaction was also observed between the *Arabidopsis thaliana* (Arabidopsis) homologs of MASH and MKB1, suggesting that MASH represents an essential and plant-specific component of this vital and conserved eukaryotic protein quality control machinery.

## Introduction

Jasmonates (JA) are oxylipin-derived phytohormones that trigger defense responses upon biotic stresses, confer tolerance to abiotic stresses, and regulate various developmental cues. One of those defense responses consists of the elicitation of the biosynthesis of bio-active specialized metabolites (Goossens et al., 2016b; Wasternack and Strnad, 2019; Lacchini and Goossens, 2020). Triterpene saponins (TSs), like those found in the model legume *Medicago truncatula*, represent one such class of defense compounds (Szakiel et al., 2011; Gholami et al., 2014) and comprise a diverse set of amphipathic molecules made up of a lipophilic aglycone backbone covalently linked to one or more sugar moieties (Thimmappa et al., 2014). Tss are derived from 2,3-oxidosqualene, which corresponds to the last common precursor between the (taxa-specific) TS and conserved sterol biosynthesis pathways (Hemmerlin et al., 2003; Thimmappa et al., 2014). TS-specific 2,3-oxidosqualene cyclases (OSCs) cyclize 2,3-oxidosqualene, which can subsequently be followed by additional modifications, mainly oxidations, by cytochrome P450s (P450s), yielding a myriad of triterpene backbones or the sapogenins. These scaffolds can be additionally decorated by UDP-dependent glycosyltransferases (UGTs), thereby further diversifying this class of specialized metabolites (Seki et al., 2015; Cárdenas et al., 2019).

Plants can produce TSs constitutively as phytoanticipins, e.g., by accumulating them in the vacuole where they reside until further bio-activation upon herbivory or pathogen attack (VanEtten et al., 1994). Conversely, the biosynthesis of certain TSs can be boosted upon predation, thus as phytoalexins, whereas some can have a bifaceted role (Pedras and Yaya, 2015). JA-triggered defense responses have been well studied in plants, and the perception and early signaling components have been well characterized (De Geyter et al., 2012; Wasternack and Hause, 2013; Chini et al., 2016; Goossens et al., 2016b; Wasternack and Strnad, 2019). Downstream of the conserved core JA signaling complex, numerous transcription factors (TFs) act to activate the expression of genes encoding enzymes that catalyze the biosynthesis of the often taxa-specific specialized metabolites (De Geyter et al., 2012; Chezem and Clay, 2016; Goossens et al., 2016b; Zhou and Memelink, 2016; Colinas and Goossens, 2018). In case of the *M. truncatula* TSs, the first and hitherto only discovered specific JA-modulated transcriptional regulators correspond to the bHLH-type triterpene saponin biosynthesis activating regulator 1 (TSAR1) and TSAR2 TFs, which were found to control, respectively, the non-hemolytic and hemolytic branch of *M. truncatula* TS biosynthesis (Mertens et al., 2016a). Recently, a seed-specific TSAR TF, TSAR3, was identified that controls hemolytic saponin biosynthesis specifically in developing *M. truncatula* seeds (Ribeiro et al., 2020). TSAR homologs were also found in *Chenopodium quinoa* and *Glycyrrhiza uralensis* to control, respectively, anti-nutritional TS and soyasaponin biosynthesis (Jarvis et al., 2017; Tamura et al., 2018), and even in the medicinal plant *Catharanthus roseus*, in which they were found to steer the monoterpenoid branch of the monoterpenoid indole alkaloid pathway but not the endogenous triterpene pathways (Van Moerkercke et al., 2015; Mertens et al., 2016b; Van Moerkercke et al., 2016).

In the mevalonate (MVA) pathway, which supplies the isopentenyl pyrophosphate building blocks for 2,3-oxidosqualene, the endoplasmic reticulum (ER) membrane-localized 3-hydroxy-3-methylglutaryl-CoA reductase (HMGR) acts as a rate-limiting enzyme. Consequently, research on the regulatory control of triterpene biosynthesis, not only in plants, has often focused on HMGR (Hemmerlin et al., 2003; Burg and Espenshade, 2011; Wangeline et al., 2017; Erffelinck and Goossens, 2018; Johnson and DeBose-Boyd, 2018). For instance, in *M. truncatula*, TS biosynthesis and the expression of the corresponding genes are also controlled by the TSARs (Mertens et al., 2016a). Because all eukaryotes produce triterpenes, more particularly at least the essential sterols, many features of HMGR regulation are conserved. Nonetheless, in some cases, specific mechanisms have evolved to allow the organism to cope with particular needs (Li et al., 2014). As such, the human genome encodes only one HMGR isoform (HsHMGR), while the genome of *Saccharomyces cerevisiae* encodes two HMGR isozymes, ScHMG1P and ScHMG2P (Burg and Espenshade, 2011). In all studied plant species, HMGR is encoded by a multigene family (Li et al., 2014). For HsHMGR and ScHMGP2, it has been reported that post-translational control is carried out by proteasomal degradation mediated by the ER-associated degradation (ERAD) machinery, the same machinery that targets misfolded proteins in the ER for ubiquitination and subsequent degradation (Burg and Espenshade, 2011; Wangeline et al., 2017; Johnson and DeBose-Boyd, 2018). The N-terminal membrane domain of HsHMGR and ScHMGP2 encompasses five consecutive transmembrane spans that constitute a sterol-sensing domain (SSD), enabling the perception of lipid signals and transmitting subsequent regulatory cues (Irisawa et al., 2009; Theesfeld et al., 2011). In mammals, 24,25-dihydrolanosterol or oxysterol trigger binding of the ER-retention protein INSIG-1 to the SSD, which accelerates HsHMGR-regulated degradation (HRD) by an ERAD machinery that involves the E3 ubiquitin ligase GP78 (Song et al., 2005; Lee et al., 2006; Tsai et al., 2012). Similarly, in yeast, terpene signals, such as geranylgeranyl pyrophosphate, can stimulate ScHMG2P turnover through an INSIG-independent ERAD machinery that involves the GP78 homolog HMGR degradation 1 (HRD1) (Garza et al., 2009; Wangeline and Hampton, 2018). HMGR in plants is structurally different from HMGR in yeast and mammals in that its membrane domain consists only of two transmembrane domains and consequently lacks the SSD (Basson et al., 1988). Additionally, because plants do not encode INSIG-1 or INSIG-1-like orthologs (Pollier et al., 2013), it is likely that plants evolved a specific mechanism to control HMGR stability. Indeed, in support of that, in *M. truncatula*, a member of a class of E3 ubiquitin ligases other than those to which the GP78/HRD1 orthologs belong, namely, MAKIBISHI1 (MKB1), was discovered, which recruits the ERAD machinery to regulate HMGR levels and thereby its activity in this species (Pollier et al., 2013). MKB1 is a so-called RING membrane-anchor (RMA)-type E3 ubiquitin ligase, which is conserved in plants and animals and has been shown to be involved in ERAD-mediated protein quality control in these organisms (Hirsch et al., 2009). The MKB1-dependent ERAD system monitors *M. truncatula* TS biosynthesis and was found to safeguard root development given that *MKB1*-silenced hairy root lines show dramatic phenotypic defects (Pollier et al., 2013). However, contrary to the analogous triterpene-regulating systems from yeast and mammalians, little is known about how the MKB1-dependent ERAD machinery operates. Plant-specific terpene or lipid signals that would trigger MKB1-dependent HMGR degradation remain elusive, as well as plant-specific mediator proteins such as INSIG analogs that mediate HMGR-MKB1 interaction, or chaperones, like analogs of HRD3, which stabilizes the HRD1 E3 ubiquitin ligase in yeast and is thereby crucial for its activity (Vashistha et al., 2016). Uncovering such elements will be paramount to understand the plant-specific control of HMGR protein levels and activity in particular, and the control of terpene biosynthesis and/or protein quality in general.

To fill these vital gaps in our knowledge, we have launched a yeast-based protein–protein interaction screen using MKB1 as bait. This allowed us to identify additional members of the MKB1-dependent ERAD machinery in *M. truncatula*, namely, an E2 ubiquitin-conjugating (UBC) enzyme, which was found capable of transferring activated ubiquitin from E1 ubiquitin-activating enzymes to MKB1, and a heat-shock protein 40 (HSP40), which supports the functioning of the MKB1 protein.

## Materials and Methods

### Cloning of DNA Constructs

Sequences of the full-length ORFs were obtained from the *M. truncatula* genome v4.0 (Tang et al., 2014). Employing Gateway^TM^ technology (Invitrogen), PCR-amplified full-length ORFs were recombined into the donor vector pDONR221. Sequence-verified entry clones were recombined with the destination vector pK7WG2D for overexpression and pK7GWIWG2(II) for silencing in hairy roots (Karimi et al., 2007). All primers used for cloning are reported in Supplementary Table 1.

### Y2H Screening and Assays

The bait vector was obtained by cloning the truncated version of MKB1 (MKB1ΔC: AA1-AA237) in the pGBT9 vector. As the prey cDNA library, we opted for a previously generated library from root nodules of *M. truncatula* A17 inoculated with a *Sinorhizobium meliloti* strain (Baudin et al., 2015). Screening of the library was performed by transformation of the PJ69-4A yeast strain with the bait by the PEG-LiAc method, subsequently super-transforming this bait strain with the Y2H cDNA library and plating on synthetic defined media devoid of Leu, Trp, and His. PCR was performed on prey plasmids of all transformants on the selective plates using vector-specific primers. After PCR purification (GeneJET PCR Purification; Thermo Scientific^TM^), amplicons were subjected to Sanger sequencing for identification of potential interactors of the bait MKB1ΔC. A complete overview of identified candidate interactors of MKB1ΔC with their gene identity and annotation is provided in Supplementary Table 2.

Subsequent Y2H assays were performed essentially as described (Cuéllar Pérez et al., 2013). Bait and prey were fused to the GAL4 activation domain or GAL4 DNA-binding domain via cloning into the pGAL424gate/pDEST22 or pGBT9gate/pDEST32, respectively (Cuéllar Pérez et al., 2013). Yeast transformants were selected on synthetic defined (SD) medium lacking Leu and Trp (Clontech, Saint -Germain-en-Laye, France). For Y3H analysis, a construct with a third potential interaction partner, N-terminally fused to a nuclear localization signal was generated by cloning into the pMG426-NLS vector (Nagels Durand et al., 2012), which was subsequently co-transformed with the bait and prey constructs. For Y3H, transformants were selected on SD medium lacking Leu, Trp, and Ura (Clontech, Saint -Germain-en-Laye, France). For both Y2H and Y3H assays, three individual colonies were grown overnight in liquid cultures at 30°C, and 10- or 100-fold dilutions were dropped on control and selective media lacking His in addition to the plasmid auxotrophy markers (Clontech).

### Phylogenetic Analysis

The E2 UBCs of Arabidopsis were collected from Kraft et al. (2005). From clade VI, E2 UBCs of *H. sapiens* and *S. cerevisiae* were also selected together with the *M. truncatula* E2 UBC Medtr3g062450. Protein sequences were aligned with ClustalW. The phylogenetic tree was generated in MEGA7 software (Kumar et al., 2016), by the neighbor-joining method (Saitou and Nei, 1987), and bootstrapping was done with 1,000 replicates. The evolutionary distances were computed using the JTT matrix-based method and are in the units of the number of amino acid substitutions per site (Jones et al., 1992). The analysis involved 41 amino acid sequences. All positions containing gaps and missing data were eliminated. There was a total of 112 positions in the final dataset. Evolutionary analyses were conducted in MEGA7 (Kumar et al., 2016).

### Generation of *M. truncatula* Hairy Roots

Sterilization of *M. truncatula* seeds (ecotype Jemalong J5), transformation of seedlings by *Agrobacterium rhizogenes* (strain LBA 9402/12), and the subsequent generation of hairy roots were carried out as described previously (Pollier et al., 2011). Hairy roots were cultivated for 21 days in liquid medium prior to sampling for RNA, protein, and metabolite extraction.

### Quantitative Reverse Transcription PCR

One hundred milligrams of frozen roots of three independent transgenic lines were ground in a Retsch MM300 mixer, and total RNA was extracted using the Qiagen RNeasy kit (Qiagen). One microgram of RNA was used for cDNA synthesis using the iScript^TM^ cDNA Synthesis Kit (Bio-Rad). qRT-PCR was performed in the LightCycler 480 System (Roche) using the Fast Start SYBR Green I PCR mix (Roche) via the following program: pre-incubation (95°C, 10 s), 45 amplification cycles (incubation 95°C, 10 s; annealing 65°C, 15 s; elongation 72°C, 15 s). Relative expression levels using multiple reference genes were calculated using qBase (Hellemans et al., 2007). The *M. truncatula 40S ribosomal protein S8* and *translation elongation factor 1a* were used as reference genes. Primer sequences are presented in Supplementary Table 1.

### Ubiquitination Assay

Recombinant glutathione S-transferase (GST)–MKB1ΔC fusion proteins [truncated with RING mutation (MKB1ΔCmRING) or without mutation (MKB1ΔC)] were purified according to the manufacturer’s instructions with Glutathione Sepharose 4B resin columns (GEHealthcare) from transformed *E. coli* cells, pretreated for 2 h with isopropyl-b-D-1-thiogalactopyranoside (IPTG). A protein refolding step to assure the full ion Zn charge of the GST–MKB1 fusion proteins was included by incubation with refolding buffer (20 mM HEPES, pH 7.4, 0.02 mM ZnCl_2_, 1.5 mM MgCl_2_, 150 mM KCl, 0.2 mM EDTA, 20% glycerol, 0.05% Triton X-100) for 1 h at 4°C. Ubiquitination reactions were performed in a total volume of 30 ml using 15 ml of the refolded GST–MKB1 bound to glutathione resin. The reaction contained 300 ng of GST–MKB1 fusion protein as E3 ubiquitin ligase, 250 ng of the ubiquitin-activating enzyme (UBE1) from rabbit (BostonBiochem), 400 ng of human recombinant UBCH5A protein (BostonBiochem) or C-terminally 6xHis-tagged Medtr3g062450 from the pDEST17 vector, and 2 mg of HA-Ub from human (BostonBiochem) in ubiquitination buffer (50 mM HEPES, pH 7.4, 2 mM ATP, 5 mM MgCl_2_, 2 mM DTT, 0.02 mM ZnCl_2_). The ubiquitination reactions were incubated for 1 h at 30°C and stopped by adding Laemmli buffer (4% SDS, 20% glycerol, 10% 2-mercaptoethanol, 0.004% bromophenol blue, 0.125 M Tris-HCl, pH 6.8). Samples were resolved on 8% SDS–PAGE, followed by protein immunoblot analysis with anti-HA (Qiagen) and anti-GST (GE Healthcare) antibodies.

### Confocal Microscopy Analysis

For co-localization analysis, the ORFs of *MKB1*, *MASH*, and *Medtr3g062450*, including and lacking their stop codon, were cloned in pDONR221 to obtain entry clones that were subsequently used to generate *CaMV35S* promoter-driven C-terminal and N-terminal GFP fusion constructs in pFAST-R05 and pFAST-R06 destination vectors, respectively, via single LR Gateway^TM^ (Invitrogen) reactions (Shimada et al., 2010).

For agro-infiltration, wild-type tobacco (*Nicotiana benthamiana*) plants were grown for 3–4 weeks. Tobacco infiltration of lower epidermal leaf cells with the *Agrobacterium tumefaciens* strains was performed as described in Boruc et al. (2010).

Image acquisition was obtained with a Zeiss 710 inverted confocal microscope, equipped with a 63× water-corrected objective (n.a. 1.2) using the following settings for EGFP and mCHERRY detection: EGFP excitation at 488 nm, emission filter 500–530 nm, mCHERRY excitation at 559 nm, and emission filter of 630–660 nm. Confocal images were acquired using the ZEN software package attached to the confocal system. Confocal images were processed with ImageJ^1^.

### Determination of 3-Hydroxy-3-Methylglutaryl-CoA Reductase Protein Levels

Protein extraction from *M. truncatula* hairy roots and determination of HMGR protein levels by immunoblot analysis was carried out as described (Pollier et al., 2013).

### Metabolite Profiling

*M. truncatula* hairy roots (five biological repeats of three independent transgenic lines per transgene construct) were grown for 21 days in liquid medium and upon harvest immediately frozen in liquid nitrogen. Processing and metabolite extraction from 400 mg of the hairy root tissue was performed as described (Pollier et al., 2011). LC-ESI-FT-ICR-MS analysis was carried out using an Acquity UPLC BEH C18 column (150 × 2.1 mm, 1.7 mm; Waters, Waltham, MA, United States) mounted on an LC system consisting of an Accela pump and autosampler (Thermo Electron Corporation, Waltham, MA, United States) coupled to an LTQ FT Ultra (Thermo Electron Corporation) via an electrospray ionization source operated in negative mode. A gradient was run using acidified (0.1% formic acid) water:acetonitrile (99:1, v/v; solvent A) and acetonitrile:water (99:1, v/v; solvent B): 0 min, 5% B; 30 min, 55% B; and 35 min, 100% B. The injection volume was 10 μl, the flow rate 300 ml/min, and the column temperature 40°C. Negative ionization was obtained with a capillary temperature of 150°C, sheath gas of 25 (arbitrary units), auxiliary gas of 3 (arbitrary units), and a spray voltage of 4.5 kV. Full MS spectra between m/z 120 and 1,400 were recorded. MSn spectra (MS2 and two dependent MS3 scan events, in which the two most abundant daughter ions were fragmented) were generated from the most abundant ion of each full MS scan. The collision energy was set at 35%. The resulting chromatograms were integrated and aligned using the Progenesis QI software (Waters). The PCA was performed with MetaboAnalyst 4.0 with Pareto-scaled mass spectrometry data and standard settings^2^ (Chong et al., 2018).

## Results

### A Protein–Protein Interaction Screen in Yeast Uncovers Novel Candidate Members of the MAKIBISHI1 Machinery

**FIGURE 1:**
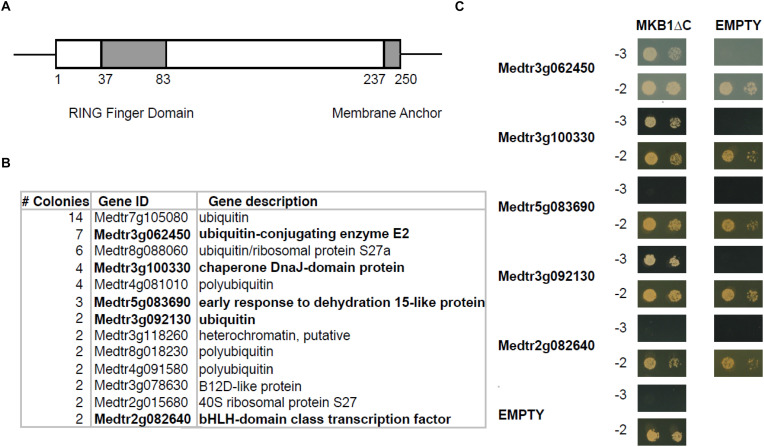
Yeast two-hybrid (Y2H) screen with MKB1ΔC. **(A)** Schematic displaying the domain organization of MAKIBISHI1 (MKB1). MKB1 contains the well-conserved RING finger domain (amino acid 37–83) and a membrane anchor (amino acid 237–250). The latter domain was removed to create MKB1ΔC. **(B)** Potential MKB1ΔC interactors identified in the Y2H screen in at least two independent transformants. The full candidate MKB1ΔC interactor list is presented in Supplementary Table 1. **(C)** Binary interaction validation of MKB1ΔC with potential interactors by Y2H. MKB1ΔC was fused to the GAL4 DNA-binding domain and full-length preys to the GAL4 activation domain. Transformed yeasts were spotted in 10- and 100-fold dilutions on control medium (–2) and selective medium (–3).

To identify novel interactors of MKB1, a yeast two-hybrid (Y2H) screen was performed using an available prey *M. truncatula* cDNA library (Baudin et al., 2015) and MKB1 devoid of its membrane spanning domain (MKB1ΔC; amino acid 1–237, to allow retrieving interactors of the catalytic domain in the Y2H system) as bait (Figure 1A). Identification of interacting preys was performed by Sanger sequencing of the respective cDNA inserts of yeast colonies that survived selection. For this study, only prey inserts identified in at least two independent transformants were considered for further in-depth analysis (Figure 1B and Supplementary Table 2). From these candidates, the full-length coding sequences were cloned *de novo* for binary interaction validation by Y2H again using MKB1ΔC as the bait. Interaction with ubiquitin, the E2 UBC and the HSP40 protein encoded by *Medtr3g092130*, *Medtr3g062450*, and *Medtr3g100330*, respectively, could be confirmed and were subjected to further analysis (Figure 1C).

### *Medtr3g062450* Encodes a Cognate Group VI E2 UBC

**FIGURE 2:**
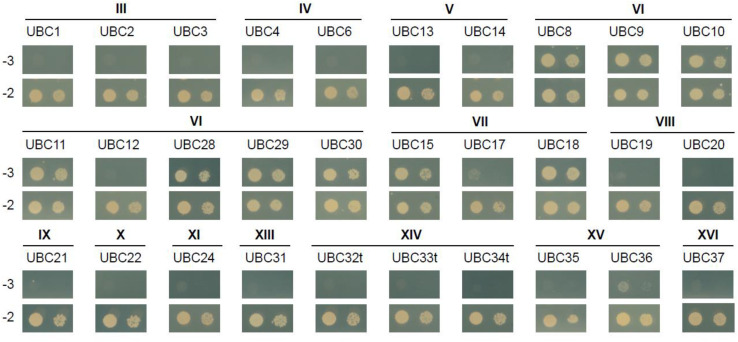
MKB1ΔC interacts with a specific subset of UBCs. Y2H assay between MKB1ΔC and a panel of 30 *Arabidopsis* E2 UBCs. The bait MKB1ΔC was fused to the GAL4 activation domain and all prey UBCs to the GAL4 DNA-binding domain. The transmembrane domain of group XIV UBCs (UBC32, UBC33, and UBC34) was removed to avoid false-negative results (named UBC32t, UBC33t, and UBC34t). Transformed yeasts were spotted in 10- and 100-fold dilutions on control medium (–2) and selective medium (–3). The different Roman numeral designations indicate E2 UBC clades (see Supplementary Figure 1).

Besides ubiquitin itself, an obvious potential additional member of the canonical MKB1 complex is the E2 UBC encoded by *Medtr3g062450*, which clusters with the clade VI E2 UBCs (Supplementary Figure 1). Group VI is the largest group of E2 UBCs comprising more promiscuous E2 UBCs that function *in vitro* with multiple E3s from different families to mediate K48-linked poly-ubiquitination, typically reported to target proteins to the proteasome for degradation, in a process called the ubiquitin–proteasome system (UPS) (Callis, 2014). Because any plant genome is predicted to encode tens of E2 UBCs, we wanted to evaluate whether MKB1ΔC uniquely interacts with E2 UBCs from clade VI. Therefore, a Y2H screen was set up using a publicly available library of Arabidopsis E2 UBCs, which contains 30 (out of 37) different E2 UBCs (Kraft et al., 2005; Nagels Durand et al., 2016). As expected, MKB1ΔC interacted with AtUBC8-11 and AtUBC28-30, which are both members of clade VI, but also with AtUBC15 and 18 that both belong to clade VII (Figure 2). Arabidopsis E2 UBCs of clade VII are related to the human UBC E2 W (Ube2W), which is reported to catalyze N-terminal ubiquitination of its target proteins (Callis, 2014). To our knowledge, to date, no specific activity or function has yet been assigned to either AtUBC15 or 18. Taken together, our interaction data show that MKB1ΔC can interact with a specific subset of E2 UBCs that are related to N-terminal ubiquitination and K48 poly-ubiquitination.

### The E2 UBC Medtr3g062450 Can Catalyze Auto-Ubiquitination of MKB1 *in vitro*

**FIGURE 3:**
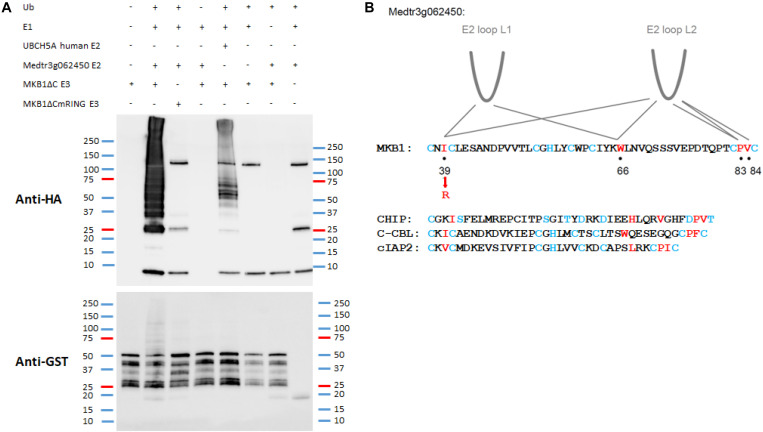
*In vitro* auto-ubiquitination assay of MKB1ΔC in the presence of the E2 UBC Medtr3g062450. **(A)** *In vitro* auto-ubiquitination assay of MKB1. The recombinant GST–MKB1ΔC protein was incubated with ATP in the presence or absence of HA-tagged ubiquitin (HA-UBQ), E1 (rabbit UBE1) and E2 UBC (human UBCH5A or Medtr3g062450). Samples were resolved by 8% SDS–PAGE, followed by protein immunoblot analysis with anti-GST. The recombinant MKB1ΔC protein possesses self-ubiquitination activity, whereas a mutated, “ligase-dead” version (MKB1ΔCmRING), in which the essential amino acid residue Ile^39^ was substituted by an Arg residue, does not. **(B)** Contacts between RING-U-box domains and E2 UBCs. RING domain sequences from the first to the last pair of zinc-binding residues for MKB1 and human CHIP, c-CBL, and cIAP2 are shown. The RING and U-box residues that make the most significant contacts observed in co-crystal structures are shown in red. The zinc-binding residues are colored in blue. The information is derived from the following co-crystal structures: CHIP:HsUBCH5, CHIP:HsUBC13, c-CBL:HsUBCH7, and cIAP2:HsUBCH5 (Zheng et al., 2000; Xu et al., 2008; Choi et al., 2009; Slotman et al., 2012).

Key structural elements of RING E3 ligases, to which also MKB1 belongs, are the two loop-like regions that coordinate the Zn^2+^ ions, surrounding a shallow groove formed by the central α-helix. Together, these elements serve as an interface for interaction with the UBC domains of E2 UBCs (Deshaies and Joazeiro, 2009; Metzger et al., 2014). To assess whether *Medtr3g062450* encodes a possible canonical E2 UBC for MKB1, an *in vitro* ubiquitination assay was performed as previously reported and in which it was shown that the human protein HsUBCH5A catalyzes auto-ubiquitination of MKB1ΔC *in vitro* (Pollier et al., 2013). We could demonstrate *in vitro* auto-ubiquitination activity of GST-tagged MKB1ΔC in the presence of recombinant His-tagged Medtr3g062450 protein and HA-tagged ubiquitin (Figure 3A). Furthermore, we evaluated whether the auto-ubiquitination activity of MKB1ΔC by the E2 UBC is dependent on the integrity of the E3 RING domain. Several functional studies of RING E3s typically employ mutations in the zinc-binding residues to inactivate the RING domain (Deshaies and Joazeiro, 2009). However, such mutations perturb the overall RING domain structure. Therefore, we targeted MKB1 residues necessary to sustain the UBC-RING contact sites instead, determined by a sequence alignment of the RING domain of MKB1 with the conserved RING-like U-box of human CHIP and the RING domains of human c-CBL and cIAP2 (Figure 3B). The predicted contact site Ile^*AA39*^ was replaced with a charged residue, Arg^*AA39*^, in MKB1ΔC (Figure 3B) resulting in a RING-dead MKB1ΔC version (MKB1ΔCmRING) that is different from the one previously generated in Pollier et al. (2013) by substituting the Cys^37^ and Cys^40^ residues by Ser residues. The absence of ubiquitination of MKB1ΔCmRING indicates that the auto-ubiquitination activity of MKB1ΔC by Medtr3g062450 is dependent on the integrity of its RING domain (Figure 3A). These results suggest that the Medtr3g062450 E2 UBC is a canonical E2 UBC for MKB1, catalyzing auto-ubiquitination of MKB1, and, consequently, possibly also ubiquitination of MKB1 targets.

### The HSP40 Encoded by *Medtr3g100330* Is Co-expressed With MKB1 and Its Target HMGR in *Medicago truncatula*

**FIGURE 4:**
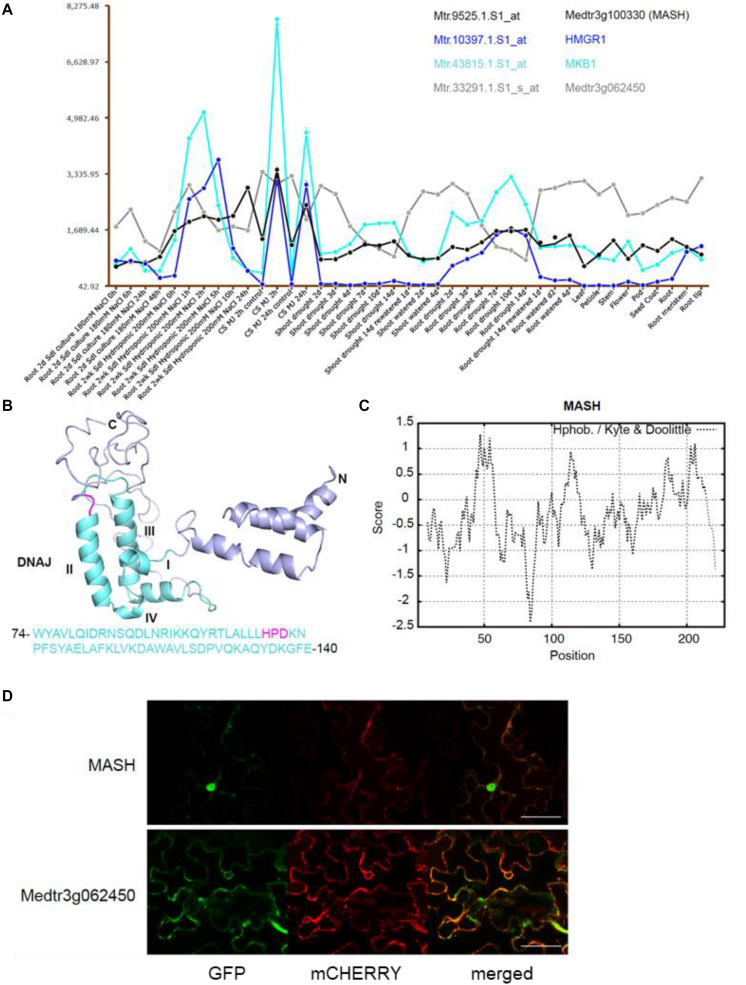
The chaperone DnaJ-domain protein-encoding gene *MASH* is co-expressed with *MKB1* and its potential target *HMGR1*. **(A)** MtGEA co-expression pattern of *MKB1* (Cyan; Mtr.43815.1.S1_at), *HMGR1* (Blue; Mtr.10397.1.S1_at), *MASH* (Black; Mtr.9525.1.S1_at), and *Medtr3g062450* (Gray; Mtr.33291.1.S1_s_at) in *M. truncatula* cell suspension cultures (CS), shoots and roots under various culturing conditions, generated with the MtGEA tool (He et al., 2009). Values in the *y*-axis represent transcript levels as stored in the MtGEA tool. MJ, Methyl JA. **(B)** Prediction of the secondary structure of MASH by Phyre with the corresponding amino acid sequence of the DnaJ domain (Kelley et al., 2015). The DnaJ domain consisting of four helices I-IV and a critical HPD signature motif are marked in cyan and magenta, respectively. **(C)** Kyte and Doolittle hydrophobicity plot of MASH, window size 15. No hydrophobic transmembrane domains were identified. **(D)** Localization of Medtr3g062450 and MASH. Confocal microscopy analysis of *N. benthamiana* leaves agro-infiltrated with constructs expressing an ER-marker fused to mCHERRY, and C-terminally GFP-tagged versions of MASH (MASH-GFP) or Medtr3g062450 (Medtr3g062450-GFP). Left to right: green, GFP fluorescence; red, mCHERRY fluorescence; merged, combined fluorescence from GFP and mCHERRY. Scale bars = 50 μm.

The second candidate member of the MKB1 E3 ligase complex is the HSP40 encoded by *Medtr3g100330*, which we named MKB1-supporting heat-shock protein 40 (MASH). Notably, mining of the transcriptome data available on the *Medicago truncatula* Gene Expression Atlas (MtGEA) (He et al., 2009) indicated that *MASH* expression was highly correlated with that of *MKB1* and its target *HMGR1* (Figure 4A). For instance, a concerted upregulation of these three genes is observed in *M. truncatula* cell suspension cultures upon methyl JA (MeJA) treatment, in roots and shoots upon drought stress and in root hydroponic systems in high-salt conditions. Expression of *Medtr3g062450* is not co-regulated with these three genes (Figure 4A), which may correspond to its plausible pleiotropic role as E2 UBC in other, MKB1-independent UPS processes. Based on its domain organization, MASH belongs to the subtype III of HSP40s that possess a canonical J-domain (Figure 4B) and generally act as obligate HSP70 co-chaperones that assist in diverse processes of cellular protein metabolism (Misselwitz et al., 1998; Laufen et al., 1999; Fan et al., 2003; Walsh et al., 2004; Craig et al., 2006; Rajan and D’Silva, 2009; Kampinga and Craig, 2010). The structure of the J-domain is conserved across all kingdoms and consists of four helices with a tightly packed helix II and III in antiparallel orientation. A flexible loop containing a highly conserved and functionally critical HPD signature motif, pivotal to trigger ATPase activity of HSP70s, connects both helices (Figure 4B; Laufen et al., 1999; Walsh et al., 2004). Hydrophobicity analysis of MASH revealed that it does not encompass a clear trans-membrane domain, indicating that it would not reside in the ER membrane as its potential ER membrane-anchored partner MKB1, but possibly is active in the cytoplasm to which also the catalytic part of MKB1 is exposed (Figure 4C). This was confirmed by co-localization studies in Agro-infiltrated *N. benthamiana* leaves, in which MASH predominantly showed a nucleocytosolic localization, whereas the E2 UBC Medtr3g062450 showed both nucleocytosolic and ER localization (Figure 4D). Co-expression of free MKB1 did not alter MASH localization either (Supplementary Figure 2). This result is not surprising given our actual difficulties in visualizing or detecting GFP-tagged MKB1 protein in Agro-infiltrated *N. benthamiana* leaves, either in the wild-type or ring-dead version. An MKB1-GFP signal was rarely visible, even in the co-localization assays; hence, we could not robustly determine its localization in this set-up. Accordingly, bimolecular fluorescence complementation (BiFC) experiments to detect *in planta* interaction between MKB1 and either MASH or E2 UBC Medtr3g062450 all consistently failed. Attempts to express and visualize GFP-tagged MKB1 protein in stably transformed *M. truncatula* hairy root lines were not successful either.

### Silencing of *MASH* Mimics the *MKB1* Phenotype

**FIGURE 5:**
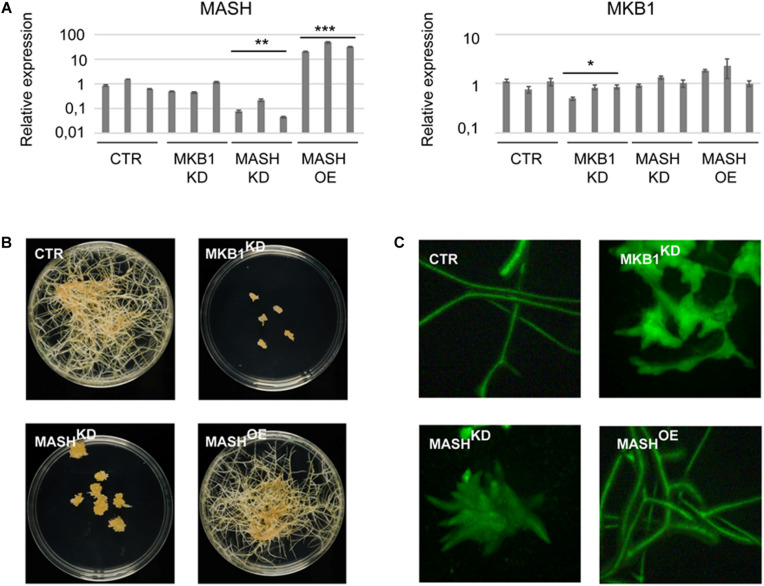
*MASH* silencing causes the “Makibishi” Phenotype. **(A)** RT-qPCR of the *MASH* and *MKB1* genes. Values in the *y*-axis represent the expression ratio relative to the mean transcript levels of the three CTR lines. Error bars ± s.e.m. (*n* = 3 technical repeats for each of the three biological repeats, i.e., the three independent transformed hairy root lines). Statistical significance between the mean of the three biological repeats was calculated by Student’s *t*-test (*P < 0.05; **P < 0.01; ***P < 0.001). **(B)** Representative images of stably transformed *M. truncatula* hairy roots (∼14 days) with control (CTR), *MASH* overexpressing (MASH^*OE*^) and *MKB1* (MKB1^*KD*^) and *MASH* (MASH^*KD*^) knock-down constructs, grown on solid medium. **(C)** Representative images of fluorescence microscopy of *GFP* expression in CTR, MKB1^*KD*^, MASH^*KD*^, and MASH^*OE*^ roots (∼14 days) grown in liquid medium. In all lines, GFP-fluorescence is derived from the expression of the *prolD-eGFP* expression cassette on the pK7WG2D or pK7GWIWG2(II) vectors.

To determine the physiological role and relevance of MASH in the MKB1 E3 ligase complex, a functional analysis was carried out *in planta*. To this end, three independent stable *MASH* overexpression (MASH^*OE*^), *MASH* knock-down (MASH^*KD*^), *MKB1* knock-down (MKB1^*KD*^), and *GUS* overexpression (CTR) *M. truncatula* hairy root lines were generated (Figure 5A). MASH^*KD*^ roots displayed a strikingly similar phenotype to that of MKB1^*KD*^ hairy roots, as previously reported by Pollier et al. (2013; Figure 5B). At the morphological level, MASH^*KD*^ and MKD1^*KD*^ roots both showed dissociation of hairy roots into “caltrop”-like structures (Figure 5C). Comparable phenotypes were not observed in MASH^*OE*^ roots (Figures 5B,C), correlating with the previously described absence of a phenotype in MKB1^*OE*^ roots (Pollier et al., 2013).

**FIGURE 6:**
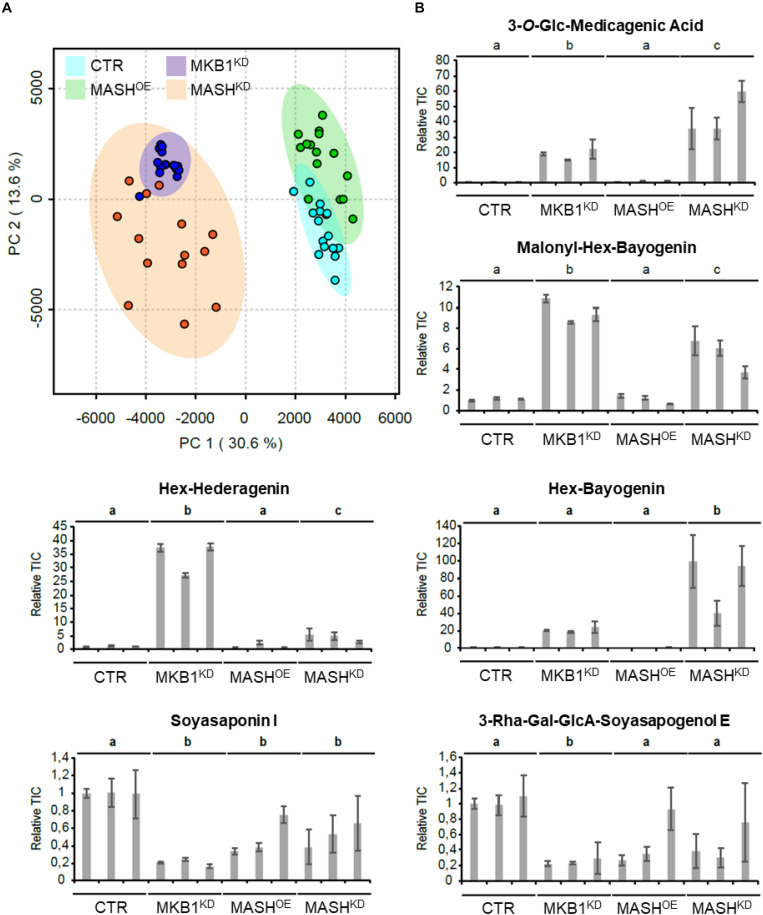
MKB1^*KD*^ and MASH^*KD*^ hairy roots have a similar metabolic phenotype. **(A)** Principal component analysis (PCA) scores plot projecting the first and second principal components of the LC-ESI-FT-ICR-MS dataset resulting from MKB1^*KD*^ (blue), CTR (cyan), MASH^*KD*^ (orange), and MASH^*OE*^ (green) roots. The samples derived from MKB1^*KD*^ and MASH^*KD*^ roots group together and are separated from the samples derived from CTR and MASH^*OE*^ roots, implying a similar metabolic profile of the MKB1^*KD*^ and MASH^*KD*^ roots. **(B)** Average total ion current (relative to line CTR1) of the peak corresponding to 3-*O*-Glc-medicagenic acid, Malonyl-Hex-Bayogenin, Hex-Hederagenin, Hex-Bayogenin, Soyasaponin I, and 3-Rha-Gal-GlcA-Soyasapogenol E. The error bars represent the s.e.m. (*n* = 5). Statistical differences between the lines (and compared relative to the CTR lines) were determined by ANOVA with a post-hoc Tukey’s HSD test (*P* < 0.01).

It has previously been reported that silencing of *MKB1* results in an altered metabolism, manifested in an altered flux toward TS biosynthesis. Detailed metabolic profiling of MKB1^*KD*^ hairy roots showed a higher accumulation of mono-glycosylated saponins, including 3-*O*-Glc-medicagenic acid, and reduced levels of high-level glycosylated saponins such as soyasaponin I (Pollier et al., 2013). To verify whether the MKB1^*KD*^ phenotype of the MASH^*KD*^ roots was also reflected in its metabolite composition, metabolite profiling by liquid chromatography electrospray ionization Fourier transform ion cyclotron resonance mass spectrometry (LC-ESI-FT-ICR-MS) was carried out on CTR, MASH^*KD*^, MASH^*OE*^, and MKB1^*KD*^ lines. A principal component analysis (PCA) on the LC-ESI-FT-ICR-MS dataset was carried out and revealed grouping of the samples derived from MKB1^*KD*^ and MASH^*KD*^ roots. These samples were clearly separated from the samples derived from CTR and MASH^*OE*^ roots (Figure 6A), implying a similar trend in the metabolic profile of the MKB1^*KD*^ and MASH^*KD*^ roots, and no major differences in the metabolite composition of CTR and MASH^*OE*^ roots. Relative quantification of diagnostic mono-glycosylated TSs, such as 3-*O*-Glc-medicagenic acid, in the various hairy root samples showed that these metabolites were significantly more highly abundant in both MKB1^*KD*^ and MASH^*KD*^ roots (Figure 6B). Conversely, like in MKB1^*KD*^ roots, several high-level glycosylated TSs, such as soyasaponin I, were significantly less abundant in MASH^*KD*^ roots (Figure 6B). Although there were still significant differences in the levels of these TSs between MKB1^*KD*^ and MASH^*KD*^ roots, it could be concluded that the trends in the alterations at the metabolite level in MKB1^*KD*^ and MASH^*KD*^ roots were similar. No significant differences between CTR and MASH^*OE*^ roots were observed for these metabolites, except for soyasaponin I (Figure 6B).

**FIGURE 7:**
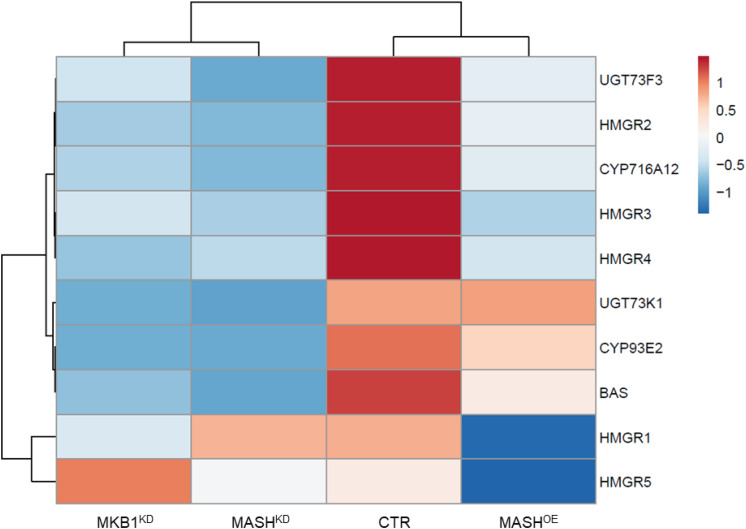
Quantitative reverse transcription PCR (RT-qPCR) analysis of TS genes in control (CTR), MKB1^*KD*^, MASH^*KD*^, and MASH^*OE*^ Roots. Heat map generated through ClustVis showing differentially expressed TS biosynthesis genes in CTR, MKB1^*KD*^, MASH^*KD*^, and MASH^*OE*^ roots. Rows are centered; unit variance scaling is applied to rows. Both rows and columns are clustered using correlation distance and average linkage. Exact expression values are given in Supplementary Table 3.

Finally, MKB1^*KD*^ hairy roots have been shown to also exert a TS-specific negative feedback on the transcriptional level (Pollier et al., 2013). To evaluate whether MASH^*KD*^ roots showed a similar transcript profile, quantitative reverse transcription PCR (RT-qPCR) was performed on TS-specific biosynthesis genes in MASH^*KD*^, MKB1^*KD*^, MASH^*OE*^, and CTR roots. Expression of the *BAS*, *CYP93E2*, *CYP716A12*, *UGT73F3*, and *UGT73K1* genes, all encoding TS-specific enzymes, was strongly downregulated in MASH^*KD*^ roots, similar to MKB1^*KD*^ roots (Figure 7 and Supplementary Table 3). Conversely, we did not detect a general downregulation of sterol-specific biosynthesis genes in MASH^*KD*^ roots, in accordance with what was observed in MKB1^*KD*^ roots (Supplementary Figure 3). Finally, and importantly, *MKB1* transcript levels were not downregulated in the MASH^*KD*^ roots (Figure 5A), supporting that the observed MASH^*KD*^ phenotypes can be attributed to *MASH* silencing and are not caused by a mere downregulation of *MKB1*. Hence, together, these data indicate that silencing of *MASH* does not affect the transcriptional regulation of triterpenes in general, but specifically affects TS biosynthesis, as is the case with *MKB1* silencing (Pollier et al., 2013).

### MASH Does Not Affect HMGR Levels *in planta*

Given that all observable MKB1^*KD*^ phenotypes were mirrored in the MASH^*KD*^ roots, we hypothesized that loss of MASH function would also affect the targeted degradation of HMGR by MKB1 in *M. truncatula*. To investigate whether MASH indeed assists in the MKB1-mediated degradation of HMGR, we monitored both HMGR transcript and protein levels *in planta* in MASH^*KD*^ roots, as well as in MKB1^*KD*^ and CTR roots, following MeJA application. The rationale behind this experimental design is that we had previously shown that effects of *MKB1* silencing on *in planta* HMGR protein levels could only be detected after MeJA application (Pollier et al., 2013). Indeed, as confirmed in the analysis conducted here, MeJA-induced *HMGR* transcript upregulation, which is observed in all lines (Supplementary Figure 4A), only resulted in detectably higher HMGR protein levels in MeJA-elicited MKB1^*KD*^ roots but not in control roots (Supplementary Figures 4B–E). Unexpectedly, however, HMGR protein levels did not significantly increase in MASH^*KD*^ roots following MeJA treatment, and showed a similar trend as the CTR lines (Supplementary Figure 4D), suggesting that degradation of HMGR may not be significantly perturbed by loss of MASH function.

In mammalian and yeast cells, the INSIG proteins bridge the ERAD E3 ubiquitin ligases GP78 and HRD1 to their targets, including the HMGR proteins (Burg and Espenshade, 2011; Wangeline et al., 2017; Johnson and DeBose-Boyd, 2018). This interaction is dependent on the membrane spanning SSD, which is absent in plant HMGR proteins. Nonetheless, we wanted to assess whether MASH can recruit *M. truncatula* HMGR1 to the MKB1 machinery and thus act as an INSIG-analog, but then as a cytosolic version that would connect the catalytic domains of HMGR1 and MKB1, which are both exposed to the cytosol. A similar function has been reported for cytosolic HSP40-type chaperones in the degradation of membrane-localized hepatic P450s (Kim et al., 2016). To this end, a Y2H assay was performed to explore the potential binary interaction between MASH and *M. truncatula* HMGR1 devoid of its membrane domain (HMGR1ΔN). However, no interaction was detected (Supplementary Figure 5A). Next, we hypothesized that MASH may only bind HMGR1 in the presence of MKB1. Therefore, a yeast three-hybrid (Y3H) assay was performed. However, also here, no interaction was observed between MKB1ΔC and HMGR1ΔN in the presence of MASH (Supplementary Figure 5B). In conclusion, our data do not support a possible role for MASH as a mediator of MKB1-HMGR interaction, at least not on its own.

### The Arabidopsis Homologs of MASH and MKB1 Also Interact

**FIGURE 8:**
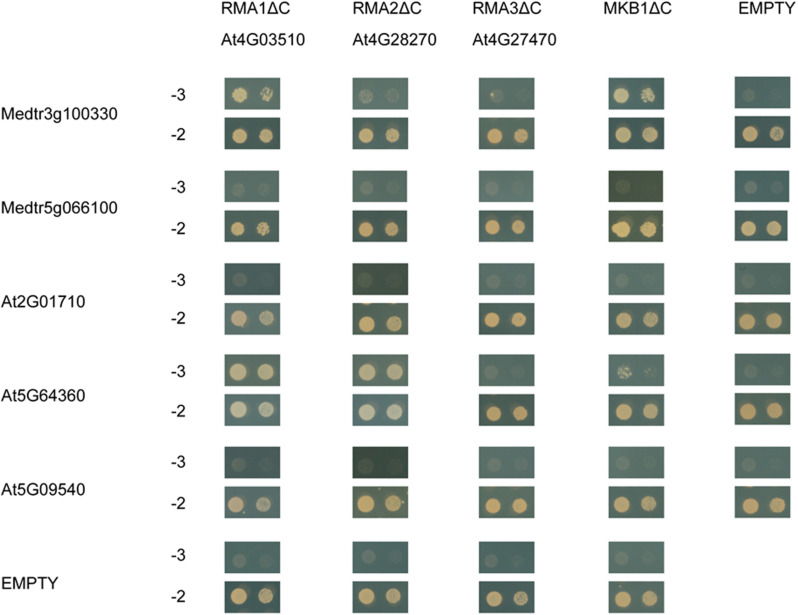
Y2H assay between MKB1ΔC and MASH homologs in *M. truncatula* and *Arabidopsis*. MKB1 or its *Arabidopsis* homologs RMA1-3, all devoid of membrane-spanning domains (MKB1ΔC and RMA1-3ΔC), were fused to the GAL4 DNA-binding domain as bait. MASH, its closest homolog in *M. truncatula* encoded by *Medtr5g066100*, and its Arabidopsis homologs encoded by *At2G01710*, *At5G09540*, and *At5G64360*, were fused to the GAL4 activation domain as prey. Transformed yeasts were spotted in 10- and 100-fold dilutions on control medium (–2) and selective medium (–3).

To assess whether MASH-MKB1 interaction may be conserved in other plant species, we assessed the interaction between the Arabidopsis RMA-type E3 ubiquitin ligase homologs of MKB1, called RMA1 to RMA3, against MASH, its closest homolog in *M. truncatula*, as well as the closest Arabidopsis MASH homologs. Arabidopsis RMAs have been previously reported as ERAD-type E3 ubiquitin ligases with possible roles in growth and development (Matsuda et al., 2001; Lee et al., 2009; Son et al., 2009). Only the Arabidopsis RMA1 devoid of its membrane domain, RMA1ΔC, could indeed directly interact with the *M. truncatula* MASH (Figure 8). The closest homolog of MASH in *M. truncatula*, encoded by *Medtr5g066100*, did not interact with MKB1ΔC, nor with any of the Arabidopsis RMAs. Therefore, interaction between MASH and MKB1 seems specific, and no redundancy seems to exist for MASH functioning in *M. truncatula*, as also evidenced by the strong phenotype of the MASH^*KD*^ roots.

Next, direct binding between the truncated Arabidopsis RMAs, MKB1ΔC, and the three closest Arabidopsis MASH homologs, encoded by *At2G01710*, *At5G09540*, and *At5G64360*, was assessed. These three homologs were identified through the PLAZA tool (Van Bel et al., 2018) and also corresponded to the first three BlastP hits for MASH in the Arabidopsis genome. We could observe specific interaction between AT5G64360 and RMA1ΔC, RMA2ΔC and MKB1ΔC (Figure 8). Together, these data suggest that a putative role of MASH in the RMA-type E3 ubiquitin ligase machinery could be conserved in Arabidopsis.

## Discussion

In the model legume *M. truncatula*, it has been shown that the JA signaling machinery recruits the ERAD E3 ubiquitin ligase MKB1 to monitor TS biosynthesis by controlling the stability of the rate-limiting enzyme HMGR, as do different, but analogous, ERAD machineries to monitor sterol biosynthesis in yeast and animals (Pollier et al., 2013). To increase our molecular understanding of this protein control apparatus, a protein–protein interaction screen was carried out that allowed to identify two hitherto unknown potential components of the MKB1-dependent ERAD machinery: a canonical E2 UBC encoded by *Medtr3g062450* and the JA-inducible HSP40 protein MASH encoded by *Medtr3g100330*. Silencing of *MASH* in *M. truncatula* hairy roots resulted in a phenotype that is similar to that of MKB1^*KD*^ hairy roots on the morphological, transcriptional, and metabolite level, indicating that MASH plays an essential role in the MKB1-dependent ERAD machinery in *M. truncatula*.

### How Does the MKB1 Machinery Target Its Substrate(s) and How Many Substrates May There Be?

One of the principal aims of the Y2H screen that we launched, was to identify the “adaptor” protein that would connect MKB1 to its target(s), in particular HMGR. This was encouraged by previous findings, in particular the observation that MKB1 can target yeast HMG2P and thereby complement a *S. cerevisiae* yeast strain devoid of the HRD1 E3 ubiquitin ligase (Pollier et al., 2013). Although *M. truncatula* uses a different family of ERAD E3 ubiquitin ligases from those directing HMGR for destruction in yeast (HRD1), they thus appeared to be compatible, suggesting that both ERAD E3 ubiquitin ligases rely on a common adaptor. Our Y2H screen revealed the HSP40 chaperone MASH as a direct MKB1 interactor, which at first sight represented an excellent candidate for a possible mediator of MKB1-HMGR interaction. Indeed, several precedents for such a role of HSP40 proteins exist in the field. In yeast, the E3 ubiquitin ligases HRD1 and DOA10 make use of an ER-resident HSP70-binding protein 3 (BiP3) to survey client ERAD substrates other than HMGR (Ruggiano et al., 2014). The DOA10 complex is also known to target ERAD substrates with lesions in the cytosolic domain, and is surveyed by the cytosolic HSP70, SSA1P, and the HSP40s, YDJ1P, and HLJ1P (Ruggiano et al., 2014). Interestingly, YDJ1P appears to be the closest homolog of MASH in *S. cerevisiae*. Likewise, in mammalian cells, an adaptor function has been suggested for cytosolic HSP40-type chaperones, again in association with HSP70 proteins, to mediate the interaction between the U-box type E3 ubiquitin ligase CHIP and its UPS target, the membrane-localized hepatic P450 protein CYP3A4 (Kim et al., 2016) or between human RMA1 and its UPS target cystic fibrosis transmembrane conductance regulator (CFTR) (Grove et al., 2011). However, our data do not support an adaptor role for the HSP40 chaperone MASH in *M. truncatula*, at least not by itself. No interaction with HMGR could be observed, nor did loss of MASH function significantly affect *in planta* HMGR levels. It is possible that the HMGR-MKB1 adaptor protein is also a membrane protein, like MKB1 and HMGR themselves, and the INSIGs. Hence, it would not have been possible to isolate it through a classical Y2H screen. Screens through other methods, such as affinity purification coupled to mass spectrometry, as we have recently established in *M. truncatula* hairy roots (Goossens et al., 2016a) and which can be adapted to isolate membrane protein complexes (Bassard et al., 2012), may offer a potent alternative, as well as the recently developed proximity labeling method with TurboID (Arora et al., 2019), which is particularly useful to detect integral membrane protein–protein interactions. Both methods may also allow revealing alternative MKB1 substrates and/or co-chaperones such as HSP70 proteins, which may reveal a multi-protein adaptor complex to bridge MKB1 and its targets.

### How Broadly Conserved Is the Role of MASH-Like Chaperones in the Support of ERAD E3 Ubiquitin Ligases?

It appears that with MASH and the clade IV E2 UBCs, *M. truncatula* MKB1 has recruited cytosolic ERAD machinery components to facilitate the degradation of ER-localized targets. Possibly this may apply to plant RMA-type ERAD E3 ubiquitin ligases in general, as evidenced by the conserved interaction between the Arabidopsis MKB1-MASH homologs. Because the Arabidopsis MKB1-homolog RMA2 can also interact with the Arabidopsis clade VI E2 UBC29 (Arabidopsis Interactome and Mapping Consortium, 2011) besides the MASH homolog AT5G64360 and because Arabidopsis RMA1 has been reported to accept ubiquitin from mammalian clade VI E2 UBCs for *in vitro* auto-ubiquitination (Matsuda et al., 2001), we postulate that the putative role of MASH in the RMA-type E3 ubiquitin ligase machinery could be conserved in Arabidopsis and possibly other (dicot) plant species as well. As an alternative to a direct role in surveying the ERAD of substrates, the interaction with MASH may aid in preserving the stability of the ERAD E3 ubiquitin ligase itself. Such a possible stabilizing role of MASH was suggested by some preliminary data. For instance, in some of our transient expression assays in Agro-infiltrated *N. benthamiana* leaves, co-expression of *MASH* with tagged *MKB1* appeared to stabilize the MKB1 protein and increase its accumulation levels. However, given the variable and low amounts of detectable tagged MKB1 protein, robust visualization by confocal imaging, or quantification of the MKB1 accumulation levels by immunoblot analysis resulted impossible; hence, strong postulation on a possible role of MASH as an MKB1-stabilizing chaperone needs more experimental support. Unfortunately, also all of our efforts to visualize or assess MKB1 protein levels in *M. truncatula* were unsuccessful, so to date, we did not manage to further probe this postulation. Nonetheless, precedents for the necessity for such a role exist in the field. In yeast, the membrane protein HRD3 is always present in a stoichiometric complex with HRD1 and is essential for the execution of HRD-dependent protein degradation because loss of HRD3 causes unrestricted self-degradation of HRD1 (Vashistha et al., 2016). An analogous system seems to exist for the multi-protein Skp/Cullin/F-box (SCF)-containing E3 ubiquitin ligase complexes. Indeed, the HSP-complex HSP90-HSP40/SGT1b was recently shown to stabilize the F-box component TRANSPORT INHIBITOR RESPONSE1 of the auxin receptor SCF complex in Arabidopsis in response to low and high temperatures to maintain proper plant growth and development (Wang et al., 2016). Research on MASH-MKB1 homologs in other plants, in parallel with further characterization of the MASH-MKB1 machinery in *M. truncatula* will allow to elucidate the functioning of this vital machinery for plant protein quality control.

## Data Availability Statement

The original contributions presented in the study are included in the article/Supplementary Material, further inquiries can be directed to the corresponding author/s.

## Author Contributions

M-LE, BR, LG, AR, JP, and AG designed the experiments. M-LE, BR, LG, AR, and JP performed the experiments. M-LE, JP, and AG analyzed the data and wrote the manuscript. All authors contributed to the article and approved the submitted version.

## Conflict of Interest

The authors declare that the research was conducted in the absence of any commercial or financial relationships that could be construed as a potential conflict of interest.

## Footnotes

1 www.imagej.nih.gov/ij

2 http://www.metaboanalyst.ca/
