# Supplementary material for: The Heat Shock Protein 40-Type Chaperone MASH Supports the Endoplasmic Reticulum-Associated Degradation E3 Ubiquitin Ligase MAKIBISHI1 in Medicago truncatula
Source: Front Plant Sci. 2021 Feb 23;12:639625. doi: 10.3389/fpls.2021.639625 (PMC7940691; doi:10.3389/fpls.2021.639625)
Supplement: Supplementary file 1 [file Data_Sheet_1.pdf]

Supplementary Material

Supplementary Table 1. Primers Used in This Study.

**Primer name**	**Primer sequence (5'-3')**
**For cloning**	
Medtr3g100330 Attb1 Fw	GGGGACAAGTTTGTACAAAAAAGCAGGCTCCATGaacacaagcaaagccgaag
Medtr3g100330 Attb2 Rv	GGGGACCACTTTGTACAAGAAAGCTGGGTCCTAtcgcttcttcgcagcag
Medtr3g062450 Attb1 Fw	GGGGACAAGTTTGTACAAAAAAGCAGGCTCCatggcatcgaaacggatcctcaaggag
Medtr3g062450 Attb2 Rv	GGGGACCACTTTGTACAAGAAAGCTGGGTCtcaacccatggcatacttctgtgtccagctg
Medtr5g083690 Attb1 Fw	GGGGACAAGTTTGTACAAAAAAGCAGGCTCCATGGAAGTTCTTTCTGCTACAAG
Medtr5g083690 Attb2 Rv	GGGGACCACTTTGTACAAGAAAGCTGGGTCTCACCTGGGCTGATGAATAGCCCG
Medtr3g092130 Attb1 Fw	GGGGACAAGTTTGTACAAAAAAGCAGGCTCCATGcagatcttcgtgaaaaccc
Medtr3g092130 Attb2 Rv	GGGGACCACTTTGTACAAGAAAGCTGGGTCCTActtgatcttcttctttgg
Medtr2g082640 Attb1 Fw	GGGGACAAGTTTGTACAAAAAAGCAGGCTCCATGgtttccccggaaaacacc
Medtr2g082640 Attb2 Rv	GGGGACCACTTTGTACAAGAAAGCTGGGTCTTAggcaactggtggacggag
Medtr3g100330 RNAi Attb1 Fw	GGGGACAAGTTTGTACAAAAAAGCAGGCTTAATGGTGTTTCGATTCCTTCTCTT
Medtr3g100330 RNAi Attb2 Rv	GGGGACCACTTTGTACAAGAAAGCTGGGTATTTTTGGTACATGCCTACATGGT
**For RT-qPCR**	
HMGR1 Fw	CAGGATTCACAGTCACAACAAC
HMGR1 Rv	GTAGACGAAGGAAGCGATGAG
HMGR2 Fw	ATCCGAACCTCTACACCTCTC
HMGR2 Rv	GCAGCAATGTCTTCTTCTTCTTC
HMGR3 Fw	ATACGACGAAGAAGATGAATC
HMGR3 Rv	CGAGTATGACGGAATTGTAC
HMGR4 Fw	TTGCTTGCCTTATCTACCTTACG
HMGR4 Rv	CACCAACAACCTCCTCATCTTC
HMGR5 Fw	CCTTAGAATCCAGACTAGCAGAC
HMGR5 Rv	CCTATCGGCATCTCACAACAC
CYP93E2 Fw	ATTGGTGAACTTCTTGGTG
CYP93E2 Rv	TCCTTCTTCCTATCACTACC
CYP716A12 Fw	AAGGGACAGCATCACCAACAC
CYP716A12 Rv	CGCCGAGATATTTGACAAGGAAAG
BAS1 Fw	AATTTCATCTCCGAGGCAGTT
BAS1 Rv	CGTGGAAGAACACATCCATCT
MKB1 Fw	CTGTGGTCACCTGTATTG
MKB1Rv	CCTGTAGTTTATTGGATTCG
Medtr3g100330 MASH Fw	TCAGATCCTCGCAATTGTTG
Medtr3g100330 MASH Rv	AGCGAGTTCGGCATAAGAAA
ELFa Fw	ACTGTGCAGTAGTACTTGGTG
ELFa Rv	AAGCTAGGAGGTATTGACAAG
40S Fw	GCCATTGTCGAATTTGATGCTG

40S Rv	TTTTCCTACCAACTTCAAAACACCG
UGT73F3 Fw	CAGCAAGTAACAGTCATCAC
UGT73F3 Rv	AGACATAGATTCAATACCTTCAG
UGT73K1 Fw	CGGATTCTTAACGCATTGTG
UGT73K1 Rv	CTCACCACTGTCTTCTTAGC
C24MT Fw	TGGATGTTGGTTGTGGTATTG
C24MT Rv	AATCAGCCTTGACGAAGTTG
CAS Fw	ACGAGGATGAGAACAGTAGATACC
CAS Rv	TGCGAGCCAGAAGTAATCAGG
CVP1 Fw	CTTAGAACCTACGCAGATATAGC
CVP1 Rv	CGCCAATAAGCAATCCTACC
CYP51G1 Fw	GACAGCACACCAGTTCAATC
CYP51G1 Rv	GCACCTATACAAGACATCCATC
CYP710A15 Fw	TTCTCTTCAATGTTGGTCTC
CYP710A15 Rv	CTCCATCTTCTCCTTACTTG
Fackel Fw	TCTCATTCACGCTCTAATTCCATC
Fackel Rv	AGAAGTCCAACCAACAGAACAAG
Hydra1 Fw	TCAAGGATAATACTGGCTTCTACC
Hydra1 Rv	TGAAACGGCAAATTGAAGGATG
SQE1 Fw	AACAACCACCGAGAACATTAC
SQE1 Rv	GTCCATCCTTGCCGAGAG
SQE2 Fw	AAGGCAACATAAGGACAATG
SQE2 Rv	ACGCATAGGCTTGAGAAG

Supplementary Table 2. Y2H Screen with MKB1ΔC as the Bait and a *Medicago truncatula* Nodule cDNA Library as Prey.

The number of independent colonies on the selective Y2H screening plates and the corresponding gene identifier and annotation for each of the identified preys, ranked by frequency, are listed.

	# Colonies	Gene ID	Annotation
1	14	Medtr7g105080	ubiquitin-60S ribosomal protein L40
2	7	Medtr3g062450	ubiquitin-conjugating enzyme E2
3	6	Medtr8g088060	ubiquitin/ribosomal protein S27a
4	4	Medtr3g100330	chaperone DnaJ-domain protein
5	4	Medtr4g081010	polyubiquitin
6	3	Medtr5g083690	early response to dehydration 15-like protein, putative
7	2	Medtr3g092130	ubiquitin-60S ribosomal protein L40
8	2	Medtr3g118260	heterochromatin, putative
9	2	Medtr8g018230	polyubiquitin
10	2	Medtr4g091580	polyubiquitin
11	2	Medtr3g078630	B12D-like protein
12	2	Medtr2g015680	40S ribosomal protein S27
13	2	Medtr2g082640	BHLH domain class transcription factor
14	1	Medtr8g007235	nucleoside diphosphate kinase
15	1	Medtr8g023040	translation initiation factor eIF-2B alpha subunit
16	1	Medtr2g009110	splicing factor 3B subunit 1
17	1	Medtr5g012210	adenine phosphoribosyltransferase, putative
18	1	Medtr7g020820	proline oxidase/dehydrogenase
19	1	Medtr7g051065	late nodulin
20	1	Medtr4g078710	ethylene-responsive transcription factor, putative
21	1	Medtr8g095390	myb domain protein
22	1	Medtr7g053330	processing peptidase beta subunit
23	1	Medtr7g027180	Nodule Cysteine-Rich (NCR) secreted peptide
24	1	Medtr4g049830	alpha/beta hydrolase family protein
25	1	Medtr1g041380	aluminum-induced-like protein
26	1	Medtr2g089815	wound-induced protein
27	1	Medtr7g116940	ubiquitin-conjugating enzyme
28	1	Medtr5g013530	jasmonate zim-domain protein 1, putative
29	1	Medtr5g064240	UDP-glycosyltransferase superfamily protein
30	1	Medtr7g113680	nuclear transcription factor Y subunit C
31	1	Medtr2g103303	embryo-specific protein 3
32	1	Medtr3g054090	transducin family protein
33	1	Medtr3g464330	microsomal omega-3 fatty acid desaturase
34	1	Medtr2g069050	elongation factor 2
35	1	Medtr8g088060	ubiquitin/ribosomal protein S27a
36	1	Medtr2g008220	UDP-glycosyltransferase superfamily protein
37	1	Medtr4g088485	polyubiquitin
38	1	Medtr7g088990	single-stranded nucleic acid binding R3H protein
39	1	Medtr2g090060	formiminotransferase-cyclodeaminase, putative
40	1	Medtr3g012420	nodulin MtN21/EamA-like transporter family protein
41	1	Medtr7g114750	DNA-damage-repair/toleration protein DRT102, putative
42	1	Medtr8g463280	homogentisate 1,2-dioxygenase
43	1	Medtr1g039380	serine/threonine-protein phosphatase
44	1	Medtr3g035130	acyl CoA ligase
45	1	Medtr2g006590	TCP family transcription factor
46	1	Medtr5g013520	jasmonate-zim-domain protein 1, putative
47	1	Medtr8g011350	serine carboxypeptidase, putative
48	1	Medtr2g435590	ethylene responsive factor, putative
49	1	Medtr3g095210	COV1-like protein

50	1	Medtr2g069310	elongation factor 2
51	1	Medtr5g013420	histone-lysine N-methyltransferase
52	1	Medtr1g082660	nuclear transcription factor Y subunit C
53	1	Medtr3g084820	nodule Cysteine-Rich (NCR) secreted peptide
54	1	Medtr5g020840	inorganic pyrophosphatase
55	1	Medtr4g064570	zinc finger protein, putative
56	1	Medtr2g020240	beta-amylase
57	1	Medtr6g026810	histidine triad nucleotide-binding protein

Supplementary Table 3. Quantitative Reverse Transcription PCR (RT-qPCR) Analysis of TS Genes in Control, $MKB1^{KD}$, $MASH^{KD}$ and $MASH^{OE}$ Roots. Indicated is the mean of gene expression relative to the normalized transcript levels of CTR line 1. ± s.e.m. (*n* = 3 technical repeats for each of the three biological repeats, i.e. the three independent transformed hairy root lines). Statistical significance was calculated by Student's *t*-test (*, P < 0.05; **, P < 0.01; ***, P < 0.001).

Gene	Genotype	Mean of relative gene expression	s.e.m.	P-value	Significance
**CYP93E2**	CTR	0,938492063	0,062261905		
	$MKB1^{KD}$	0,061480159	0,003403571	0,000580689	***
	$MASH^{KD}$	0,047698413	0,003468254	0,000499509	***
	$MASH^{OE}$	0,687301587	0,037142857	0,161795404	
**CYP716A12**	CTR	1,212184874	0,1017507		
	$MKB1^{KD}$	0,283193277	0,039929972	0,001101035	**
	$MASH^{KD}$	0,183123249	0,0217507	0,000848701	***
	$MASH^{OE}$	0,441876751	0,071638655	0,00519054	**
**UGT73F3**	CTR	1,18486424	0,150491046		
	$MKB1^{KD}$	0,472559214	0,05413056	0,008682163	**
	$MASH^{KD}$	0,296187175	0,023298671	0,0045733	**
	$MASH^{OE}$	0,559214327	0,064818024	0,013439216	*
**UGT73K1**	CTR	0,927095077	0,050807967		
	$MKB1^{KD}$	0,136264562	0,013776776	0,000120984	***
	$MASH^{KD}$	0,082863585	0,004047351	2,88243E-05	***
	$MASH^{OE}$	0,933859451	0,063773018	0,931710415	
**BAS**	CTR	1,45490982	0,136740147		
	$MKB1^{KD}$	0,426853707	0,077020708	0,003759708	**
	$MASH^{KD}$	0,327989312	0,022371409	0,001972141	**
	$MASH^{OE}$	0,920507682	0,077955912	0,086560229	
**HMGR1**	CTR	1,395039322	0,216212946		
	$MKB1^{KD}$	0,915910466	0,216515426	0,041518909	*
	$MASH^{KD}$	1,378705384	0,159407139	0,946601826	
	$MASH^{OE}$	0,453115547	0,113793103	0,002179593	**
**HMGR2**	CTR	1,495351926	0,263612218		
	$MKB1^{KD}$	0,389110226	0,101062417	0,01920281	*
	$MASH^{KD}$	0,309030544	0,047941567	0,01653573	*
	$MASH^{OE}$	0,657104914	0,110358566	0,057286953	
**HMGR3**	CTR	1,208037825	0,111300236		
	$MKB1^{KD}$	0,707801418	0,052529551	0,004834309	**
	$MASH^{KD}$	0,651536643	0,034089835	0,002485723	**
	$MASH^{OE}$	0,660992908	0,040236407	0,001549382	**
**HMGR4**	CTR	1,258724428	0,082310469		
	$MKB1^{KD}$	0,356799037	0,097352587	0,003751428	**
	$MASH^{KD}$	0,42087846	0,069855596	0,007162234	**
	$MASH^{OE}$	0,472924188	0,086161252	0,006391489	**
**HMGR5**	CTR	0,971370143	0,07091002		
	$MKB1^{KD}$	1,27402863	0,067586912	0,034340845	*
	$MASH^{KD}$	0,893149284	0,042791411	0,404721792	
	$MASH^{OE}$	0,376789366	0,02908998	0,000181135	***

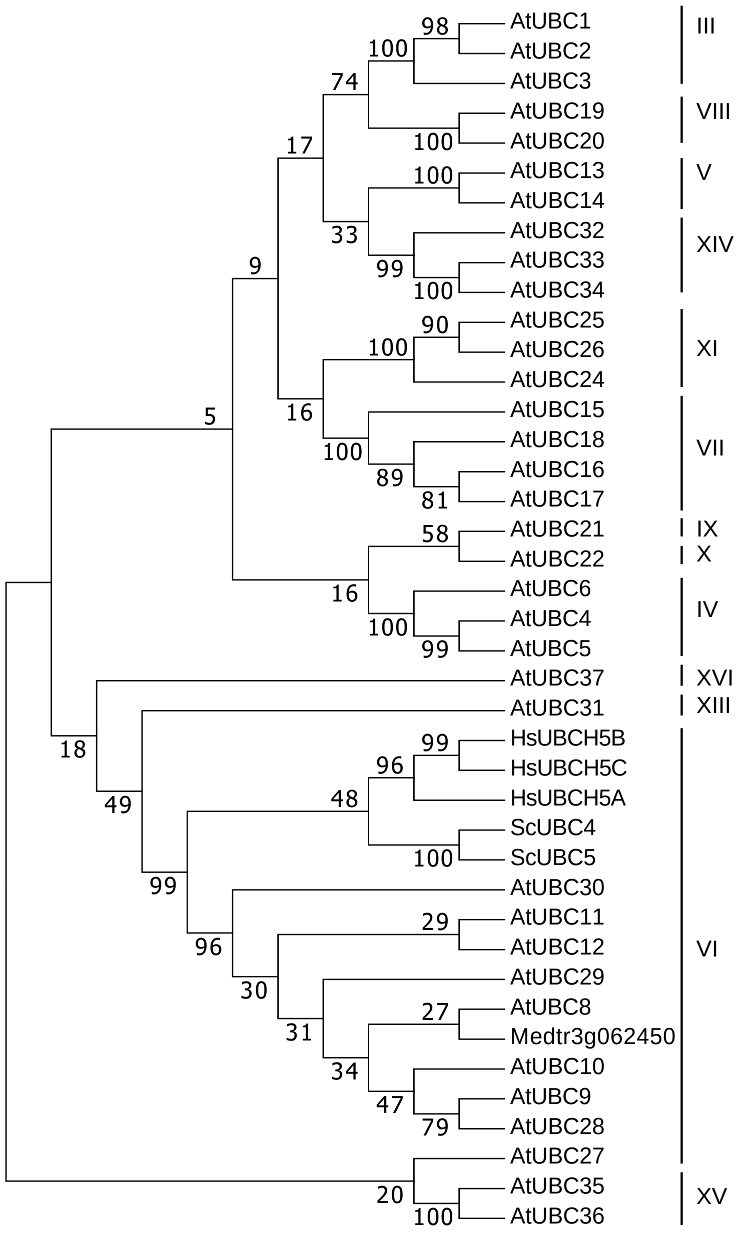

Supplementary Figure 1. Phylogenetic Tree of a Selected Set of E2 UBCs of *Arabidopsis thaliana*, *Homo sapiens*, *Saccharomyces cerevisiae* and *Medicago truncatula*.
The phylogenetic tree was generated by the neighbor-joining method and bootstrapping was done with 1000 replicates. Branches corresponding to partitions reproduced in less than 50% bootstrap replicates were collapsed. The percentage of replicate trees in which the associated taxa clustered together in the bootstrap test are shown next to the branches.

MASH-GFP +
MKB1

MASH-GFP+
MKB1mRING

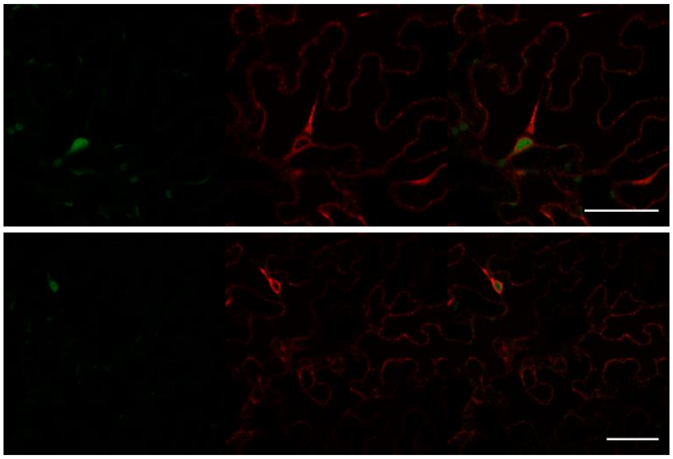

Supplementary Figure 2. Localization of MASH in the presence of MKB1 or MKB1mRING. Confocal microscopy analysis of *N. benthamiana* leaves agro-infiltrated with constructs expressing an ER-marker fused to mCHERRY, MASH (MASH-GFP), MKB1 (without a tag) or MKB1 mRING (without a tag). Left to right: green, GFP fluorescence; red, mCHERRY fluorescence, merged, combined fluorescence from GFP and mCHERRY. Scale bars = 50 μm.

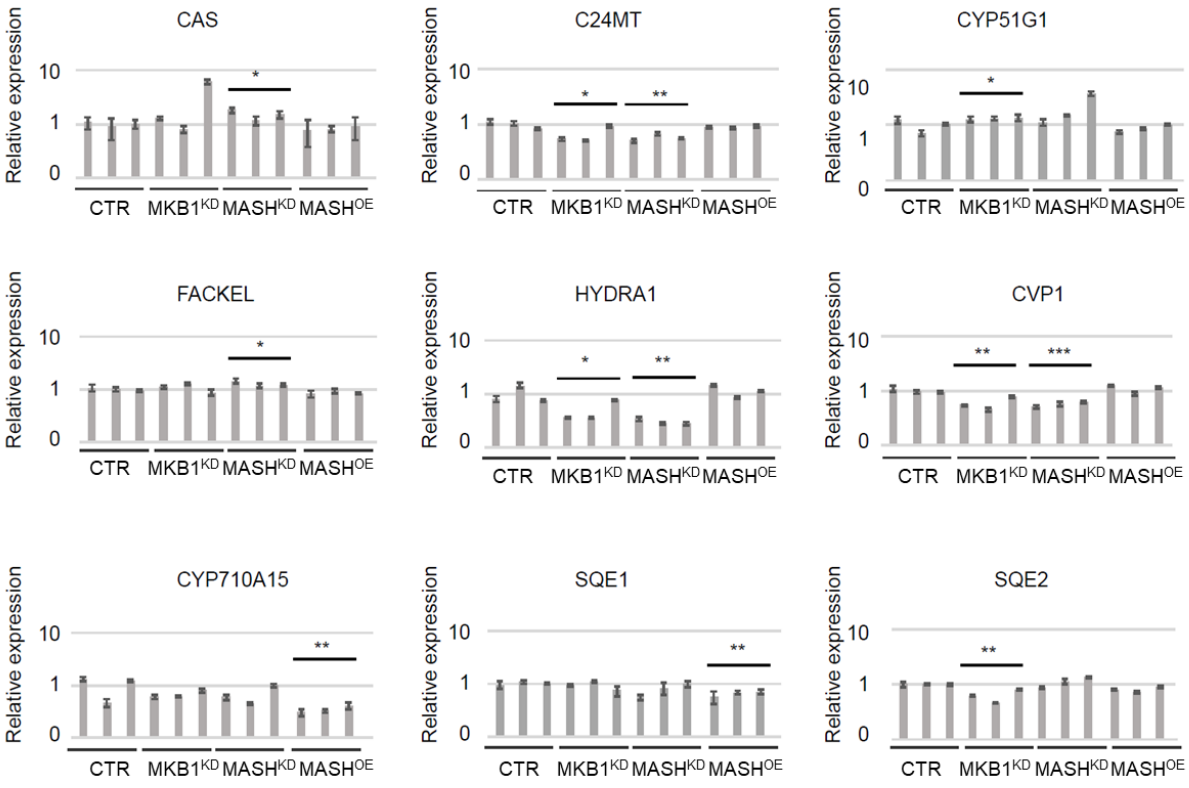

Supplementary Figure 3. qPCR Analysis of Sterol Biosynthesis Genes in CTR, MKB1^{KD}, MASH^{KD} and MASH^{OE} Roots.

Values in the y-axis represent the expression ratio relative to the mean transcript levels of the three CTR lines. C24MT, C-24 methyltransferase; CAS, cycloartenol synthase; SQE, squalene epoxidase; SQS, squalene synthase; CVP1, cotyledon vascular pattern1. Error bars, ± s.e.m. (n = 3 technical repeats for each of the three biological repeats, i.e. the three independent transformed hairy root lines). Statistical significance between the mean of the three biological repeats was calculated by Student’s t-test (*, $P < 0.05$; **, $P < 0.01$; ***, $P < 0.001$).

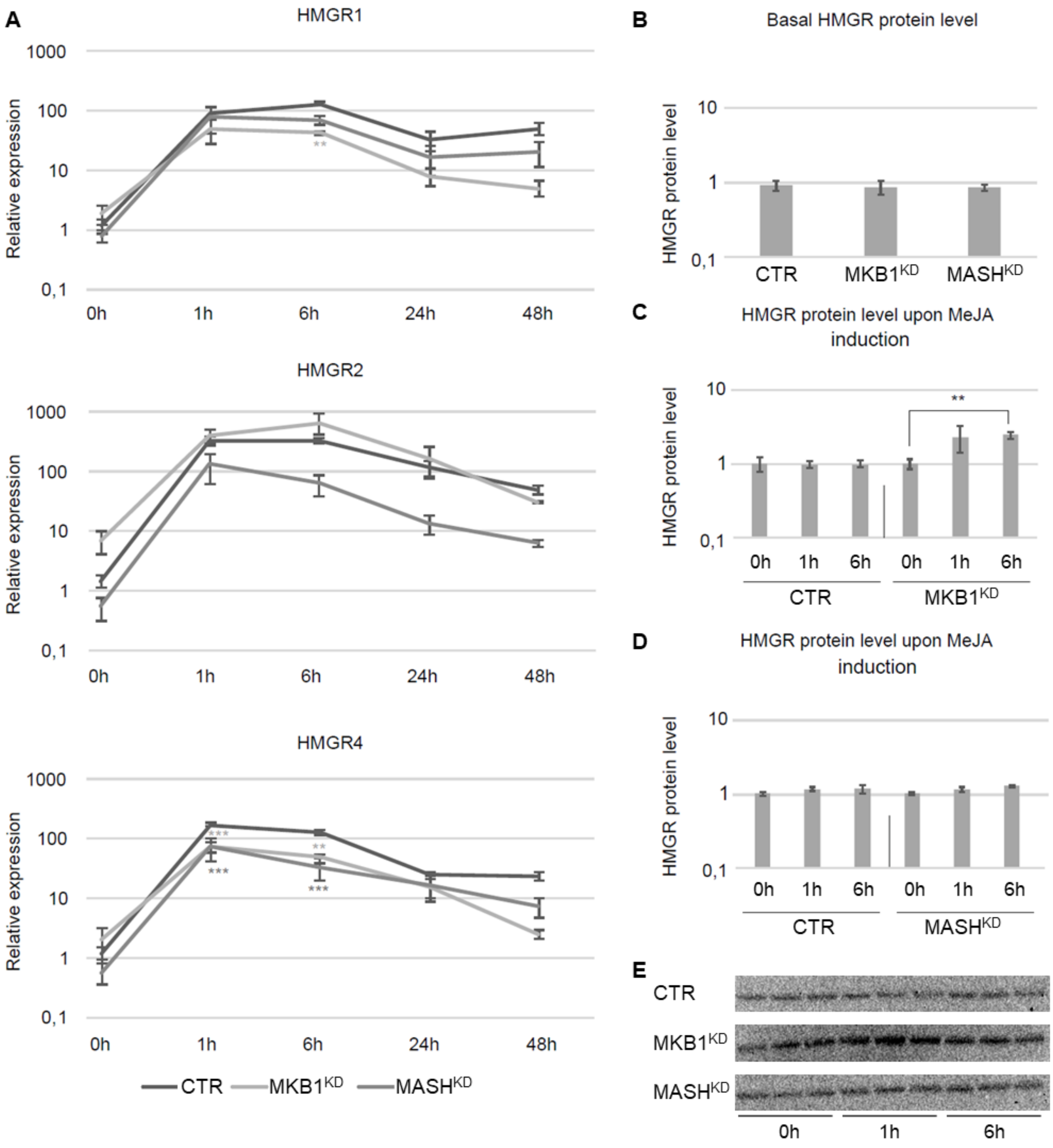

Supplementary Figure 4. HMGR Expression in MeJA-Treated CTR, MKB1^{KD} and MASH^{KD} Roots.

(A) RT-qPCR analysis of *HMGR* transcript levels in CTR, MKB1^{KD} and MASH^{KD} hairy roots treated with MeJA. Error bars, ± s.e.m. ($n = 3$ independent transformed hairy root lines, each measured in 3 technical repeats). Two-way ANOVA with post hoc Tukey was performed to assess effect of MeJA-treatment on *HMGR* levels in CTR, MKB1^{KD} and MASH^{KD} roots (*, $P < 0.05$; **, $P < 0.01$; ***, $P < 0.001$; statistical significance between the mean of the three biological repeats). Transcript levels of *M. truncatula HMGR1*, *2* and *4* reached a maximum between one to six h of MeJA treatment in all lines, i.e. CTR, MASH^{KD} and MKB1^{KD} roots. **(B)** HMGR protein levels in mock-treated hairy roots. No significant differences are observed between lines. **(C)** HMGR protein levels in MeJA-elicited

CTR and MKB1^{KD} roots. HMGR protein levels in MeJA-elicited MKB1^{KD} roots increased significantly by ca. 2.5-fold after 6 hours (h). No such increase is visible in MeJA-elicited CTR roots. **(D)** HMGR protein levels in MeJA-elicited CTR and MASH^{KD} roots. No significant differences are observed following MeJA-treatment in the MASH^{KD} roots. HMGR protein levels were quantified by immunoblot analysis. Values in the *y*-axis of (B-D) represent the ratio of HMGR protein levels relative to the normalized levels of the mock at 0 h. Error bars, ± s.e.m. ($n = 3$ independent transformed hairy root lines). Statistical significance was calculated by Student's *t*-test (*, $P < 0.05$; **, $P < 0.01$; ***, $P < 0.001$). **(E)** Representative immunoblots for the analysis of HMGR protein levels shown in **(B-D)**.

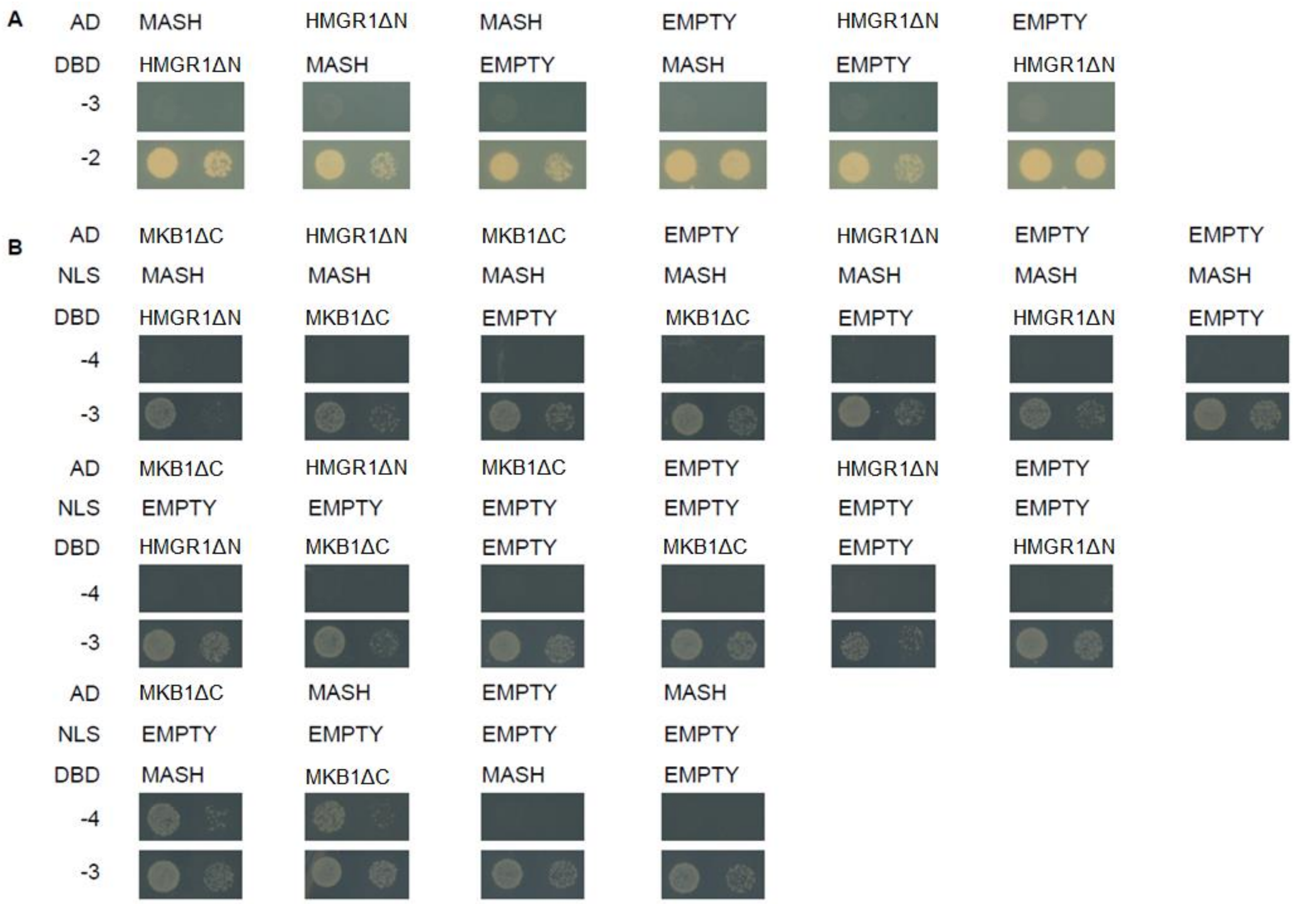

Supplementary Figure 5. Y2H and Y3H Analysis with MKB1ΔC, HMGR1ΔN and MASH.
(A) MKB1ΔC and MASH were fused to the GAL4 activation domain or GAL4 DNA-binding domain and expressed in the PJ69-4A yeast strain. Transformed yeasts were spotted in 10-fold and 100-fold dilutions on control medium (-2) and selective medium (-3).
(B) MKB1ΔC and HMGR1ΔN were fused to the GAL4 activation domain or GAL4 DNA-binding domain and together with the N-terminal nuclear localization signal (NLS)-fused MASH expressed in the PJ69-4A yeast strain. Transformed yeasts were spotted in 10-fold and 100-fold dilutions on control medium (-3) and selective medium (-4).
